# Redesigning the model for providing primary healthcare in Iran: a grounded theory study in rural settings

**DOI:** 10.1017/S1463423626101194

**Published:** 2026-06-03

**Authors:** Fereshteh Fani, Ghahraman Mahmoudi, Jamshid Yazdani-Charati, Seyedeh-Niko Hashemi, Mansooreh Gharayagh-Zandi, Seyed Amir Soltani, Mousa Yaminfirooz, Mohammad-Ali Jahani

**Affiliations:** 1 Islamic Azad University Sari Branch, Islamic Republic of Iran; 2 Department of health services administration, Sar. C., Islamic Azad University Sari Branch, Islamic Republic of Iran; 3 Biostatistics Department, School of Health, Health Sciences Research Center, Mazandaran University of Medical Sciences, Islamic Republic of Iran; 4 Shiraz Medical School: Shiraz University of Medical Sciences, Islamic Republic of Iran; 5 Ph.D Candidate of health services administration, Sar.C, Islamic Azad University Sari Branch, sari, Islamic Republic of Iran; 6 Babol University of Medical Science, Islamic Republic of Iran; 7 Social Determinants of Health Research Center Health Research Institute, Babol University of Medical Science, Islamic Republic of Iran

**Keywords:** healthcare providers, Iran, primary health care, rural health services

## Abstract

**Aim:**

This study aimed to redesign the model for providing primary healthcare (PHC) in rural Iran using a grounded theory approach.

**Background:**

Iran’s PHC system, although historically successful, faces structural, managerial, and demographic challenges that limit its responsiveness to emerging population health needs. A context-sensitive redesign is therefore required.

**Methods:**

This applied qualitative study used grounded theory methodology. Semi-structured interviews were conducted with 25 senior PHC managers, policymakers, and academic experts selected through purposive and theoretical sampling. Data collection and analysis proceeded concurrently using open, axial, and selective coding in MAXQDA 20. PCAT dimensions were used as sensitizing concepts, while allowing new categories to emerge inductively.

**Findings:**

The core category identified was ‘strengthening PHC through integrated service delivery and system enablement’. The redesigned model includes core service delivery components (utilization, access, continuity, coordination, comprehensiveness, and affiliation with place/provider) and enabling system components (evidence-based planning, managerial capacity strengthening, financing and payment management, effective surveillance and supervision). These enabling components function as structural supports sustaining effective PHC delivery in rural Iran.

## Introduction

After bringing many achievements in the early years of implementation, the primary healthcare (PHC) system in Iran has faced various challenges in recent years. The failure to implement corrective interventions and make essential changes in the structure and processes of the PHC system is a significant cause of these challenges. These changes should align with the evolving conditions and needs of the country (Khankeh *et al*., 2021). In recent years, demographic changes and increased life expectancy has resulted in increased demand for health services. People are more aware of their health needs, and with improved economic situations and increased ability to pay, this demand has risen even further (Damari *et al*., 2019). These factors, along with induced demand, have contributed to the growing consumption of health services, resulting in increased costs for the health system (Malakoane *et al.*, 2020). In addition, the fragmentation of health systems, injustices in people’s access to health services, and the focus on treatment and specialized health services have impacted the efficiency of health systems (Tilford, 2018).

In 1978, the Alma Ata conference was held with the aim of presenting a strategy to strengthen health systems. PHC was introduced as the most important strategy to strengthen health systems and achieve the goal of universal health coverage (Tilford, 2018). This strategy was less commonly used worldwide due to insufficient financial resources and countries moving towards market-oriented and treatment-based health systems. Despite recognized systemic obstacles, countries were urged to strengthen PHC as a foundation for health system reform (World Health Organization, 2000). After a period of stagnation in the shadow of financial concerns for health, in 2008, Primary care should serve as the central coordinating point of national health systems, enabling services to be organized around people’s health needs and delivered in a people-centred manner (World Health Organization, 2008). In later years, the WHO focused on a single programme that reflected concerns about equity and justice. Based on this, the strategy of universal health coverage became the motto of this organization to realize justice and human rights (Rifkin, 2018). Formulating sustainable development goals was the most important international action to achieve this goal, based on which all the member countries of the United Nations agreed to try to achieve universal health coverage by 2030 (Tsimtsiou, 2017). Achieving universal health coverage requires strengthening PHC rather than merely expanding facilities (Tsimtsiou, 2017). However, the goal of universal health coverage cannot be achieved only through the development of health facilities; rather, implementing the PHC system with an emphasis on comprehensiveness, quality, society orientation, and cost-effectiveness of services is essential to achieve this goal (Tashiro and Kohsaka, 2021).

Global policy guidance emphasizes the central role of primary care in coordinating health systems (World Health Organization, 2008); this approach enables the health systems to support the health needs of people and provide healthcare in a way that focuses on people’s needs. Also, this approach is vital to strengthen the resilience of health systems against critical conditions Strengthening PHC is essential for improving resilience and advancing universal health coverage goals (World Health Organization, 2020). More recently, global policy discourse has been reframed through the Astana Declaration and subsequent operational frameworks, which emphasize PHC resilience, equity, digital transformation, and universal health coverage acceleration (World Health Organization, 2018; World Health Organization, 2020). These contemporary frameworks move beyond structural reform toward system integration and adaptive governance models. This issue has caused health system policymakers around the world to look for an effective model to provide PHC and treatment effectively.

In 1985, Iran established health and treatment networks to address the needs of society. The effort aimed to achieve the desired goals through three main components: setting up healthcare centres in remote and sparsely populated villages, employing health workers from the local community (called Behvarz), and creating a simple but fully integrated health information system (Mehryar, 2004). Reducing the mortality of mothers and infants, increasing vaccination coverage, eradicating some communicable diseases, active care for non-communicable diseases, increasing the health awareness of community members, and improving health indicators have been the most important achievements of this plan in the early years of its implementation (Mehryar, 2004; Malekafzali, 2009). Gavaghan *et al.* in Australia (Gavaghan *et al*., 2024), Kpamma *et al.* in Ghana (Kpamma *et al.*, 2023), and Bi and Liu in England (Bi and Liu, 2023) emphasized the need to change from traditional healthcare by conducting research in the field of transforming and improving the PHC delivery process. They emphasized the value-based and evidence-based process that allows for integration at all levels of care and the need for leadership at all levels for active support. In Iran, the research of Azimzadeh *et al.* (Azimzadeh *et al*., 2023), Doshmangir *et al.* (Doshmangir *et al*., 2023), and Rezapour *et al.* (Rezapour *et al.*, 2019) also presented solutions to create innovative changes in response to new fields and emerging challenges.

With the change in social, economic, and cultural conditions, as well as the epidemiological changes of diseases, the demands and expectations of society have changed (Sajadi and Majdzadeh, 2019). Despite significant achievements, Iran’s PHC system lacks the necessary flexibility to meet the emerging needs of society due to changes in demand and the burden of diseases. This may seriously affect the health system (Mehryar, 2004; Nekoei-Moghadam *et al.*, 2012). The system’s functional weaknesses are evident regarding access to services and the referral system, particularly in urban areas. There’s also a lack of continuity of care due to the absence of full-time doctors and non-compliance with emerging health issues. Additionally, systemic weaknesses exist in organization and management, financing, health information systems, and payment and incentives systems. The evidence indicates that Iran’s PHC system requires redesigning to meet society’s needs and implement intervention measures to modify the structure and processes. Therefore, this study aims to redesign the model for providing PHC in Iran.

## Methods

### Study design

This study employed a grounded theory methodology (Straussian approach) to generate a conceptual model grounded in empirical data. Grounded theory was selected because the aim was not to test an existing framework but to develop a context-sensitive model for PHC redesign in rural Iran. Data collection and analysis were conducted concurrently, allowing theoretical sampling and constant comparison to guide subsequent interviews.

The research population included key and expert people in the field of health provision, such as senior health managers, health assistants of universities of medical sciences, informed and knowledgeable doctors and managers at the national level, as well as experts in health and treatment service management, health policy and health economics.

### Reflexivity

The research team consisted of academics and health system researchers with prior experience in PHC policy and management. Their professional background may have influenced interpretation toward system-level perspectives. To mitigate bias, reflexive memos were maintained, peer debriefing sessions were conducted, and emerging categories were repeatedly compared against raw interview data.

### Setting

This study was conducted in rural regions of Iran where PHC services are primarily delivered through health houses and rural comprehensive health centres. These settings were selected due to their historical significance in Iran’s PHC model and current structural challenges.

### Participants

Participants included 25 senior PHC managers, policymakers, and academic experts with at least five years of experience in rural health system planning or implementation. Participants were selected using purposive and theoretical sampling to ensure diversity in experience and institutional affiliation.

### Data collection

Next, the interviewees were asked which components of this tool should be included in the proposed model for redesigning and strengthening the country’s PHC system. Which component is not suitable for Iran’s conditions and should be removed? And what are the other components that should be added to this model according to the country’s conditions?

PCAT dimensions were used as sensitizing concepts to guide semi-structured interviews rather than as fixed categories. Components were retained, modified, or expanded based on empirical data. The Primary Care Assessment Tool (PCAT) was developed by The Johns Hopkins Primary Care Policy Center for Underserved Populations to measure the amount and quality of primary care. It is designed in a service delivery environment designated by recipients as their primary source of general care and consistent with a focus on primary care features that have been shown to produce better outcomes of care at lower costs. According The Primary Care Assessment Tool (PCAT) was originally developed by the Johns Hopkins Primary Care Policy Center to evaluate the quality and extent of primary care services (Johns Hopkins Primary Care Policy Center, 2024).

### Data analysis

Data analysis followed the grounded theory approach through three iterative stages: open coding, axial coding, and selective coding. During selective coding, the core category – strengthening PHC through integrated service delivery and system enablement – was identified. All categories were theoretically integrated around this core concept to explain how service delivery functions and enabling system components interact to redesign PHC. To ensure the validity of the research, the interview questions were confirmed by several experts. Among the criteria for evaluating qualitative studies, we can point to trustworthiness, credibility, dependability, transferability, and conformability (Makri and Neely, 2021). In order to obtain these criteria, the following steps were taken: transcription of interviews and continuous analysis along with data collection during the interviews, checking the coding of the interviews by another expert to ensure the correctness of the coding and the accuracy of the researcher’s understanding from the subject interviews. In most qualitative research, the researcher shows and confirms the results of his analysis to the interviewees to obtain more reliability. We reached saturation after interviewing 25 experts and senior managers. After compiling the proposed initial model, to ensure the correctness of coding and determining the main and sub-components of the model, three expert panel meetings with the presence of the research team and eight experts in the field of PHC were held. For example, statements related to ‘outdated equipment’ and ‘lack of prioritization’ were initially coded as resource inadequacy (open coding), grouped under utilization challenges (axial coding), and ultimately integrated into the core category of system enablement during selective coding. Changes were made in the model if necessary.

### Framework of analysis

**Figure 1.**
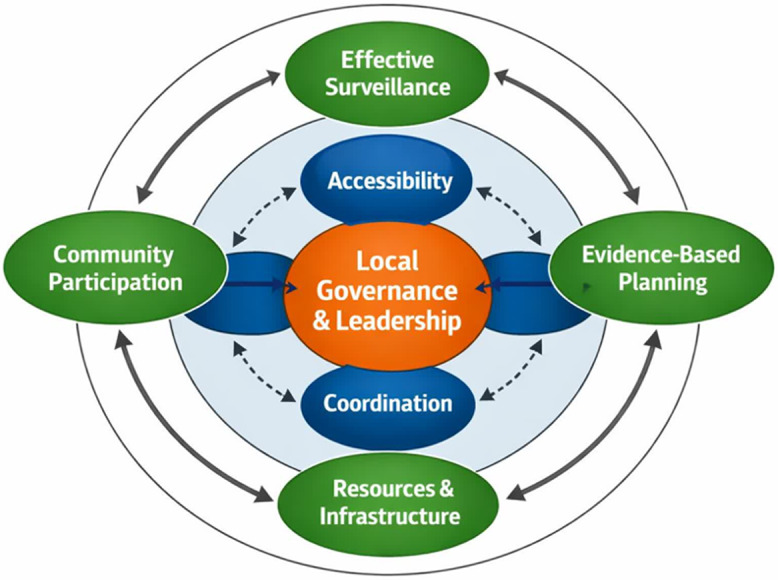
Figure 1 long description. Conceptual model of the redesigned PHC system for rural Iran.

The analytical framework was guided by grounded theory principles and informed by PCAT dimensions as sensitizing concepts. Figure 1 presents the conceptual model emerging from selective coding. The figure illustrates the interaction between core service delivery components and enabling system-level components that collectively strengthen rural PHC.

### Findings

About 6 (24%) of the interviewees were senior managers of the Ministry of Health. 14 (56%) were experts, and 14 (56%) had doctorates. Regarding work experience, the minimum was 11 years, and the maximum was 35 years (Table 1).

**Table 1.** Demographic characteristics and contextual factors of the participants Table 1 long description.

Variable	Description	Number	Percent
**Gender**	Female	18	72
	Male	7	28
**Degree**	Doctorate	14	56
	General practitioner	7	28
	Master	4	16
**Employment status**	Ministry of health and medical education	6	24
	Medical schools	14	56
	Health network	5	20

The redesigned model of providing PHC was obtained by analysing the data from the interviews. The proposed model has eight main components and 32 sub-components in two structural and process categories. It also consists of 7 supporting components with 15 sub-components (Table 2).

**Table 2.** The redesigned model: main, supporting, and sub-components from the interviews Table 2 long description.

Component		Sub-component
Main components	Utilization	Behaviour of the provider to provide primary healthcare
		Compilation of equipment identification
		Agility in providing and repairing equipment
		Attention to quality in purchasing equipment
		Attention to equipment calibration
		Reviewing and prioritizing defined equipment
	Accessibility	People’s access to health services
		Telehealth for remote healthcare centres
		Availability of infrastructure
	Continuity	Establishing a continuous relationship between the provider and the recipient of services
		Continuous retraining of Behvarz^1^ according to the needs
		Career and academic promotion of Behvarz
		Increasing the knowledge of Behvarz to work with the system
		Choosing Behvarz with high communication skills
		Choosing Behvarz with a suitable university education
		Providing necessary human resources for rural healthcare centres
		Empowering managers, trainers and staff experts
	Coordination of registration systems	Registration of health information of the covered population
		Connecting health partner organizations to an integrated electronic system
		Agility and user-friendliness of the information registration system
		Enhancing and updating the information registration system (not changing it constantly)
	Coordination between primary care system and referral system	Exchange of information between parties involved in care
		Continuous and responsible response of the information registration system support team
		Beneficiaries’ access to electronic health records at all levels of health service provision
	Comprehensiveness of services received	Providing a set of individual essential health services for the target population
		Assessment of regional needs for developing a service package
		Providing integrated services
	Comprehensiveness of services available	Integrating a set of essential individual health services into the care plan
	Affiliation with the location/service provider	The presence of a health care facility that is suitable to both the patient and the provider
		Transparency of the budget for the renovation and maintenance of the physical space
		Building safety (Health Environment Safety)
		Strengthening buildings
Supporting components	Cultural competence	Providing services according to the values, language, heritage, and common beliefs of the community
	Family-centeredness	Paying attention to family characteristics in health and psychosocial interventions
		Self-care by trained household
	Community orientation	Knowing the basic health needs of a community
		Commitment of other government institutions to provide health
		Systematization of donations from charities and non-governmental organizations
	Evidence-based planning	Using evidence for decision-making
		Regional semi-centralized and decentralized implementation (regional authority)
	Enhancing managerial thinking	Community oriented human resource training
		Strengthening collaborative management
	Financial management and payment	Financing
		Performance-based pay
	Effective surveillance	Monitoring skills
		Simultaneous monitoring of input, process, and output
		Track feedback

^1^The local healthcare worker.

### Utilization

Having equipment and necessities is one of the basic prerequisites for people to benefit from basic health services. Compilation of the equipment ID card and analysis of the existing condition of the equipment is a basic step for the management of equipment and supplies. One of the participants mentioned, *‘Currently, the health centers have outdated equipment that cannot be used. There are also instances where we are unaware of the available equipment and the required supplies’ (p1).* The solution proposed by the participants is to evaluate the current equipment using a monitoring checklist and create an equipment ID card. One participant mentioned, *‘We use a checklist for monitoring health centers. We can also have a checklist for equipment. For example, when we buy a device for you, we should track how often it was used, how efficient it was, and whether it was managed properly. We don’t have these records, and they should be compiled’ (P18).*

Ensuring the quality and safety of healthcare centres’ equipment is another factor that can strengthen the use of basic health services. A health manager at the University of Medical Sciences mentioned, *‘Currently, the health network is compelled to purchase low-quality equipment due to limited financial resources and the necessity to buy everything on credit. Sellers also supply substandard materials because they receive their payments late and incomplete’ (P5).* However, paying attention to the calibration of the equipment can solve the problems and shortcomings of the existing equipment. One of the managers of the health network believed that *‘periodic evaluation of the equipment in the health centers and their improvement is an important issue that is usually overlooked in the health centers. If the healthcare workers are trained, they can perform the calibration. Their abilities should be utilized (p4)’.*

It is necessary to identify the broken equipment, provide the required equipment, and make it available to the health service providers. Prioritizing equipment needed for purchase can improve the efficiency of equipment management. However, the lack of an equipment prioritization system is one of the shortcomings of our health network system in Iran, as mentioned by the participants. In this regard, one of the directors of the Ministry of Health believed, *‘We do not have a clear prioritization for the equipment at the first level of healthcare. We do not know which ones are priorities and which ones are not. Are these priorities provided based on local needs or not?’ ( P18).* On the other hand, strengthening the equipment supply chain is a necessary prerequisite to achieving this goal. One of the participants in this regard believed that *‘the process of requesting equipment is now long and tedious. For everything, superior managers must agree. The Behvarz (local health worker) may be tired of tracking the equipment they need. Therefore, they try to use the same worn-out equipment that works badly’. (P11).*

### Accessibility

Geographical extent and the presence of remote villages are the main factors affecting people’s access to basic health services. Residents in remote areas, who need these services the most, have little access to them. Using telehealth services can improve access to these basic health services. One of the senior managers said in this regard, *‘Telemedicine and the use of the internet for providing healthcare services should be taken seriously at health centers. Doctors working there should have access to electronic prescription equipment, and an electronic referral system should be available at the health center as well. This requires sufficient bandwidth, internet access, and a computer system with the necessary capacity’. (P8).* However, the lack of infrastructure was the main challenge mentioned by the participants in achieving this goal.

## Continuity

Continuity of services is one of the essential principles of PHC. It requires effective communication between providers and recipients of health services and information sharing between them. The existence of information-sharing infrastructure and the empowerment of human resources are among the most important prerequisites for the continuation of health services. The participants believed that strengthening the selection of human resources, retraining them, and empowering them in Iran’s PHC system face many challenges. It is vital to focus on reforming these processes.

In this regard, one of the human resources managers of the university stated, *‘If we select a nurse with good communication skills in addition to education and knowledge, it will enhance the primary care team’s interaction with people and improve interdepartmental cooperation’. (P12).* Another important point is that the selection of human resources should be appropriate to the current circumstances and also based on the competencies needed to provide services effectively. One participant stated that *‘in the past, health centers provided limited services. However, due to the current needs of society, the number of services has increased. Sometimes, there is a need to enhance the necessary skills in this regard. Health workers with lower levels of literacy may be unable to provide certain services, such as using electronic files, which requires the ability to work with this type of equipment’ (P23).*

Lack of human resources is another important challenge that can make the continuity of services difficult. Analysing the existing human resources, estimating the required number according to the volume and nature of services, and attracting human resources are the main interventions that will solve this challenge. One of the health network managers believed that *‘the Ministry of Health has announced that it will increase the services in order to provide, maintain and improve the health of the community members and according to the needs of the people. We estimated a certain number of human resources needed for the mentioned services, but we never had enough money to hire that many people. As a result, our existing employees cannot fully provide the service package, leading to problems with service continuity’. (P13).*

Training and empowering human resources, including health service providers and managers at different levels, is another solution that can strengthen the continuity of services. In most cases, the service provider initiates communication with the patient, which is the main factor in its continuity and effectiveness. Therefore, human resource empowerment is an essential prerequisite. In this regard, one of the interviewees believed that *‘we should improve the design of the health education system. It means that the quality of educational programs should be improved and educational technology should be used more’ (P25).* Another participant continued, *‘In-service courses should be evaluated. It should be determined how targeted these courses are. How much capability does it create for people taking these courses? And how much it is based on their needs. The training is sometimes one-sided; that is, we from the headquarters unit ask people to participate, get a grade, take an exam, and get a certificate. We never saw the potential of that person working in the rural parts for several years’ (P8).*

### System coordination

Recording people’s health information and sharing it in the form of electronic health records leads to a better understanding of people’s health problems and increases the effectiveness of therapeutic interventions. In addition, this system can increase the coordination of services at different levels of delivery and improve access to services. The interviewees believed that achieving this goal requires the existence of the necessary infrastructure, consolidation, and integration of information registration systems, as well as ensuring user-friendliness of the health information registration system. One of the interviewees believed that *‘creating an electronic health record requires good internet access in all regions of the country and information recording systems in all centers’ (P24).* However, the multiplicity of information registration systems has brought a serious challenge to the integrity of information. One of the participants believed that ‘Now different systems are being used at PHC centres in the country. Much of the information of those systems cannot be accessed in other systems; we have many problems in this field’ (P4).

### Coordinating the referral system

One of the most important functions of the health information system is the sharing of information between different providers during the patient referral process; the lack of information sharing reduces the continuity of services and causes confusion for patients and a lack of coordination among health service providers. The existence of an effective information system requires access to this information by all stakeholders, including service recipients, service providers at different levels, and health managers. In this regard, one of the managers of the Ministry of Health said, *‘In hospitals, we have an information registration system that is different from the healthcare center systems. Medical specialists in private offices have almost no system. The same goes for private general practitioners’ (P18).* One of the health network managers added, *‘Currently, the nurse or the health care provider should actively follow up on a diabetic patient and ask them what the specialist prescribed for them because this information is either not recorded or unavailable to the nurse’ (P17).*

### Comprehensiveness of existing and provided services (available and received)

The comprehensiveness of offered services significantly impacts the availability and continuity of services. If individuals have access to a wide range of essential services when visiting service providers, they are more likely to continue using these service units. However, the interviewees believed that achieving this goal requires accurate identification of the health needs of individuals and communities, developing a service package based on the identified needs, and aggregating these services in a single unit.

One of the participants said in this regard, *‘In Iran, we created a series of service packages without considering the actual needs of the people. Instead of asking the people what kind of services they would like to receive from us, we made assumptions based on high disease burdens in society. For example, if a certain disease, like Disease X, was prevalent, we automatically added that service to the network system. However, people may prefer to receive that specific service from another place, and we didn’t consider their preferences’. (P22).*

Another point is that after identifying the needs of people, the relevant services should be integrated into the package of basic health services. For this, it is necessary to pay attention to issues such as the ability and skill of human resources to provide these services, the equipment and supplies required, the financial resources required, and the prioritization of services to enter the package. One of the interviewees said, *‘Behvarz is expected to do everything alone, without recognition for their abilities or limitations’. (P3).* Another participant added, *‘In integrating services in the package of basic health services, we should consider how much time it will take. How many health services will be added? How much physical resources and financial resources do we need? We should take all of these into account’ (P20).*

### Affiliation with the location/service provider

Having a suitable place in terms of physical space and in terms of the existence of a care provider is one of the basic prerequisites for continuing to receive health services. The transparency of the budget for the reconstruction and maintenance of the physical space is an important and effective step for managing the maintenance of service centres. One of the participants stated in this regard, *‘We have not had construction resources for health centers for years; we used to have them, and now the health centers are very worn out, and there are no resources to rebuild them’. (p11).* Another participant mentioned, *‘Although they provided a loan many years ago for reconstruction and improvement, deprived areas still face problems. Proper allocation and effective use of loans can significantly impact the work of the Behvarz’ (P14).*

Ensuring the quality and safety of the health centre building is another factor that can strengthen the continuity of receiving the service. The participants believed that if the safety and quality standards are not met in the centres, there will be no desire for recipients to use the services and no enthusiasm for providers to provide care. One of the interviewees said, *‘There are no security boundaries where they should be. I visited a health center recently and noticed that there were no walls or any kind of barrier around the health center. It is located in a remote part of the village, and because of this, clients are afraid to come there. Sometimes, the security, protection, and peace of mind that a Behvarz (community health worker) needs for their work may not be provided’. (P15).*

The safety of the health centre depends on the building’s strength and resilience against accidents, as emphasized by the experts. One said, *‘The current topic is the safety of structures, being addressed by the Ministry of Health. The ministry is closely monitoring all units. According to evaluations of our health units, 32% were found to lack necessary preparation, either due to structural issues or other problems’. (P2).*

### Cultural competence

Health services should be in accordance with the values, language, heritage, and common beliefs of society in order to be used by them. The interviewees believed this issue is more important, especially in primary health care, which closely involves individuals and communities. Creating a degree of decentralization, considering the different background conditions of various regions can help the country’s PHC system achieve its goal. One interviewee believed, *‘Nearly all of the services within our network system are decided upon by senior managers at the Ministry of Health for the entire country. These managers may be unaware of the unique conditions and cultural differences in various regions of the country. This lack of awareness sometimes results in the announcement of programs that are not accepted by the people in certain regions’. (P10).*

### Family-centeredness

Family-centeredness is an approach to primary health care delivery in which the patient’s family and health care providers are involved in making decisions about services. Family involvement in the care process can improve care outcomes and increase patient compliance with medical orders. One of the participants said in this regard, *‘People feel more responsible when they feel that they are being noticed; this feeling of responsibility makes them help more in caring and become more interested in this process’ (P15).*

Family participation in the care process requires attention to families’ conditions, backgrounds, and needs. The interviewees believed that the family should feel that health services are personalized for them. In this case, they will be interested in participating in care. In addition, the necessary infrastructure for family participation, such as electronic health records, is essential. One of the interviewees said, *‘If we want families to participate and be able to help in self-care, all the information related to the health of that family should be recorded in one place. The family should have access to the relevant information’ (P9).*

### Community orientation

Community-oriented PHC is directed to meet the needs of a community. Doctors and PHC team members can play an important role in strengthening community-centred services by communicating with the community and departments inside and outside the health system. One of the interviewees said, *‘Participatory infrastructures, like legal infrastructures, are essential for stakeholders to participate in the health of society. For instance, Behvarz should be a member of a local council and contribute to planning regularly. When I mention that it should be a legal organization, I mean that it should be officially established through principles of council cooperation in the Ministry’. (P25).*

The participants also believed that determining the roles and duties of all institutions related to PHC, attracting the participation of non-governmental organizations, and seeking support to gain the opinion of policymakers to formulate related laws can help the health system achieve this goal.

One of them said, *‘Whenever we involve people, we achieve good results. For instance, during the COVID pandemic, we relied on people’s help to provide aid. In financial matters, too, people have participated effectively, but it’s crucial to organize this involvement to address the most important needs of society’ (P3).*

### Evidence-based planning

Achieving each of the mentioned components by the PHC system in the country requires detailed planning based on valid evidence. The existence of many and sometimes contradictory changes in health programmes is one of the important challenges that the interviewees mentioned. One of the participants believed in this regard, *‘Sometimes they announce a program or add a service, without having studied about it, this makes the health workers upset or it is boring for them. That’s why they have less commitment to implement it’ (P7).* Organizing the health information system and conducting applied research to create the necessary evidence is a prerequisite to achieving this goal. One of the interviewees stated in this regard, *‘We need to start by gathering scientific evidence, understanding the context and global perspective, benchmarking, learning from other countries’ experiences, piloting the program, implementing it, and finally, communicating it to the entire country. Unfortunately, this process is often not followed’. (P23).*

It is important to consider the differences in conditions and contexts of different regions when implementing programmes. This consideration increases the likelihood of success for health programmes by ensuring they are tailored to the specific conditions and context of their implementation. One of the participants stated in this regard, *‘The cities of our country are different in terms of social, economic, cultural factors, and even climate, ethnic and racial conditions. Every program we design must be flexible enough so it can be slightly changed based on the needs of each region. Obviously, the way pregnant mothers are cared for and the way they are informed and recruited in Sistan and Baluchistan province is different from Semnan province and East Azerbaijan province, and the way people are encouraged to have children in Hormozgan province is different from Khuzestan province and Yazd province’ (P3).*

### Enhancing managerial thinking

Strengthening management skills and creating a correct attitude towards PHC in the country is an essential intervention to strengthen PHC. One of the participants believed that *‘we train doctors who do not understand the nature and importance of primary healthcare at all, we train them for the hospital, and then we deploy them to the healthcare centers’ (P12).* In confirmation of this issue, another participant said, *‘We are not trained as primary healthcare providers at the university. From the beginning, we thought we would have an office and treat patients. But now, in primary healthcare, I must get involved with the community and communicate with institutions and organizations. I have to make plans, monitor and analyze statistics, and… I must learn these things through experience and trial and error’ (P13).*

Achieving effective results in PHC requires the participation of all stakeholders in the service delivery process. Therefore, managers, as one of the programme’s main beneficiaries, must learn the knowledge and skills of participatory management. One of the participants believed that *‘a manager in the health department should be able to attract the participation of the health workers. This participation should be both in the design of the service and its implementation and monitoring. We do not see this. The local health council was a really good idea but could not take shape because it was not supported’ (P9).* However, creating partnership structures alone is not enough. The health department managers should be empowered to carry out this work, and other stakeholders should also have the necessary ability to participate.

### Financial management and payment

In the field of financing, providing sufficient financial resources and using efficient payment methods were the most important things that the participants suggested for strengthening primary care. A major part of the payment to human resources in Iran’s PHC sector is non-motivational and has nothing to do with the individual’s performance. For example, one of the financial managers of the universities stated, *‘In the payment system, we have linked a small part of the employee’s payment to their performance. That is, the main part of the payment is fixed. Therefore, the employee receives almost the same payment regardless of their performance’ (P18).* Some participants mentioned reasons such as the absence of clear performance expectations, unreliable performance information, delayed payment deposits, and unfairness in payment as factors contributing to the ineffectiveness of the performance-based payment system in Iran.

### Effective surveillance

Effective monitoring provides the possibility of identifying the deviation of activities from the set goals. The interviewees believed that this issue requires monitoring skills, monitoring the structural, process, and outcome aspects of health programmes, and following up on monitoring feedback. One of the university managers believed that *‘we need supervisors who themselves are scientifically proficient in the subject. Sometimes, our local health workers have high knowledge of the work, which is even more proficient than the supervisors themselves’ (P14).* Another participant believed *that ‘At the same time that Behvarz is monitored, they should be taught how to solve these problems. Behvarz should feel that monitoring is for improving things’ (P16).*

Effective monitoring and supervision in PHC should be able to evaluate the determined structures, processes, and outcomes. In addition, it must be able to determine the correctness and accuracy of the information. One of the interviewees said, *‘After creating the electronic health record, we are only using the registered information of the health care providers in this system to evaluate their performance. We all know that a percentage of these registered services are unreal. It is better to have combined remote and in-person monitoring. That means the information recorded in the system must be verified in person’ (P5).*

The person responsible for monitoring should remember that the purpose of monitoring is to improve service delivery processes. To achieve this goal, feedback about the weaknesses of the service providers and the methods for correction should be provided to the person being monitored. One of the interviewees stated, *‘If the supervisor notices a weakness during monitoring, they should not just give a low grade and leave. The providers should be taught how to correct it. Until the process is corrected, monitoring should continue. When the problem is fixed, the monitoring has reached its goal’ (P17).*

Description: The model presents four core components (Accessibility, Comprehensiveness, Coordination, and Local Governance & Leadership) arranged in a central circle, with Local Governance at the centre as the integrating element. Four supportive components (Effective Surveillance, Evidence-Based Planning, Community Participation, and Resources & Infrastructure) surround the core in an outer ring. Solid arrows from supportive to core components indicate enabling relationships. Dashed bidirectional arrows among core components show interdependence. The central position of Local Governance highlights its role in coordinating and empowering all other elements.

### Discussion

The proposed model has eight main components and seven supporting components. Utilization, accessibility, continuity, coordination (care system and referral system), comprehensiveness (services received and available services), and affiliation with the place/service provider are the main components of this model. Also, cultural competence, family-centeredness, community orientation, evidence-based planning, strengthening managerial thinking, financial and payment management, and effective monitoring and supervision are the supporting components of the model. This model has a total of 32 main sub-components and 15 supporting sub-components. Several models and tools have been introduced to evaluate primary health care (Buljac-Samardzic *et al*., 2020). While PCAT has traditionally been applied as an evaluative instrument for measuring PHC performance, the present study extends its application by using PCAT dimensions as sensitizing concepts for system redesign. This approach enables the identification of both core service delivery functions and enabling system capacities required for strengthening PHC in the Iranian rural context. So far, several versions of this tool have been developed, and in various studies, researchers have tried to present a localized model of this tool according to the conditions and context of each country (D’Avila *et al.*, 2017; Aoki *et al*., 2020; Fani *et al*., 2023). Hoa *et al.* have developed a native version of this tool for Vietnam. This tool includes six main dimensions and three supporting dimensions. The six dimensions representing the main principles of (PHC include comprehensiveness (services provided, services available), ongoing care, coordination, accessibility, utilization, and first contact. This tool has three supporting dimensions, including family-centeredness, community orientation, and cultural competence (Hoa *et al.*, 2018). In another study, Yang *et al.* localized this tool to China. This tool has nine dimensions: comprehensiveness, family-centeredness and community orientation, coordination, service and communication, first contact (access), ongoing care, outreach, first contact utilization, and stability of primary care providers (Yang *et al*., 2013). The Japanese version of this tool also includes these components: first contact, being longitudinal, coordination, comprehensiveness (services provided and services available), and community orientation (Aoki *et al*., 2016).

In the present study, core PCAT components such as first contact, continuity, coordination, comprehensiveness, and utilization were retained due to their universal relevance. In contrast, components such as evidence-based planning, managerial capacity strengthening, financing and payment management, and effective surveillance emerged inductively as context-specific requirements for the Iranian PHC system and were therefore added as supporting dimensions. PCAT was developed based on the basic principles of primary care. Therefore, the principles of first contact, comprehensiveness, continuity, and coordination exist in most localized tools. However, some components have been added or removed based on the conditions and context of each country (Yang *et al*., 2013; Wang *et al*., 2014; Aoki *et al*., 2016; Harzheim *et al.*, 2019). While early WHO reports laid the conceptual groundwork for PHC reform, contemporary global frameworks place greater emphasis on measurable performance indicators, cross-sectoral governance, digital integration, and system resilience. This evolution reflects the increasing complexity of health system stewardship and reinforces the need to adapt core primary care principles to contemporary demographic and epidemiological transitions. The contextual expansion observed in the present model is therefore consistent with the global trajectory of PHC reform. It’s important to consider the specific conditions and context of each country when adapting this tool to local needs while still staying true to its core principles. This helps make the tool more compatible. Additionally, using the PCAT tool and customizing it for different countries allows for meaningful comparisons between different health systems. This is why it’s important to emphasize the use of PCAT as the primary tool for evaluating the PHC systems across the world’s countries.

In the current research, while maintaining the basic principles of this model, components that are suitable for the local conditions of Iran have been tried to be added to the model. For example, according to experts, evidence-based planning is essential for strengthening Iran’s primary health care system. Other studies have shown that paying less attention to scientific and valid evidence in health policy-making has been a major challenge for Iran’s health system (Rahimi *et al*., 2022; Doshmangir *et al*., 2023). In another study, ‘effective monitoring and supervision’ was added as a main component of the model. In the present study, the experts believed that Iran’s current monitoring and supervision system has several weaknesses, which must be eliminated to strengthen PHC in Iran. Sadegh Tabrizi, his colleagues, and Yazdi Faizabadi and his colleagues showed in their studies the weakness and ineffectiveness of monitoring and supervision in primary health care in Iran (Yazdi-Feyzabadi *et al*., 2015; Tabrizi *et al*., 2017).

This study employed the Primary Care Assessment Tool (PCAT) dimensions not merely as evaluation metrics but as sensitizing concepts to guide the systemic redesign of primary health care. While the PCAT provided a foundational framework, our grounded analysis revealed that its standard dimensions required significant contextual adaptation. Crucially, several context-specific enabling components – such as evidence-based planning, managerial capacity strengthening, financing mechanisms, and effective surveillance – emerged inductively as essential system capacities for the Iranian rural setting. This localization process mirrors adaptations seen in other countries, where PCAT-based tools have been modified to incorporate local priorities like community governance or specific payment models. The key modification in our model is the explicit elevation of these enabling, system-level components from implicit background factors to defined, actionable elements of the redesigned system.

## Limitations

This study has several potential limitations to consider when using its results. First, the participants in this research were selected using snowball sampling, and an attempt was made to invite experienced and knowledgeable people to participate in the study. However, the opinions of these people may not represent the opinions of all experts in the country. Second, the main focus of this research is on strengthening the PHC system in rural areas of the country. Therefore, due to the difference in the context of urban and rural areas, caution should be taken when generalizing the research results. Another limitation of this study is that participants were predominantly senior managers and academic experts. The absence of frontline health workers (e.g., Behvarz) and service recipients may have resulted in a top-down perspective, potentially underrepresenting experiential and community-level insights into PHC delivery.

## Conclusion

Although PCAT is primarily used as an evaluation tool for PHC, it inherently outlines the core components of an efficient primary care system. This makes it a valuable foundational model for system redesign in various countries. In this study, we utilized expert opinions to localize the PCAT tool for the Iranian context. The resulting localized, PCAT-based model proposed here can guide the systematic redesign of Iran’s PHC system. However, given the dynamic nature of Iran’s health system, future adaptations to this model may be necessary. Therefore, continuous monitoring of the PHC system and applied research are strongly recommended to adapt the model to evolving needs and contexts. These steps will assist policymakers in designing and implementing effective, evidence-based interventions.

## Data Availability

The data sets used and/or analysed during the current study are available from the corresponding author upon reasonable request.

## Figure 1. Long description

The conceptual model of the redesigned primary healthcare system for rural Iran consists of a central component labeled ‘Local Governance & Leadership’ surrounded by interconnected elements. These elements include ‘Effective Surveillance,’ ‘Evidence-Based Planning,’ ‘Resources & Infrastructure,’ ‘Community Participation,’ ‘Accessibility,’ and ‘Coordination.’ Arrows indicate the flow and interaction between these components, emphasizing the importance of local governance and leadership in managing and improving the primary healthcare system. The model highlights the need for effective surveillance, evidence-based planning, adequate resources and infrastructure, community participation, accessibility, and coordination to address the challenges faced by the healthcare system in rural Iran.

Navigate back to Figure 1..

## Table 1. Long description

The table presents demographic characteristics and contextual factors of participants. It includes three main categories: gender, degree, and employment status. For gender, there are 18 females and 7 males. For degree, 14 participants have doctorates, 7 are general practitioners, and 4 have master’s degrees. For employment status, 6 work at the Ministry of Health and medical education, 14 at medical schools, and 5 at health networks. The table provides both the number and percentage of participants in each category.

Navigate back to Table 1..

## Table 2. Long description

The table presents a detailed comparison of the main components, sub-components, and supporting components of a redesigned model for primary healthcare. It consists of eight main components and 32 sub-components divided into two categories: structural and process. Additionally, there are seven supporting components with 15 sub-components. The main components include Utilization, Accessibility, Continuity, Coordination of registration systems, Coordination between primary care system and referral system, Comprehensiveness of services received, and Comprehensiveness of services available. Each main component is further broken down into specific sub-components. The supporting components include Cultural competence, Family-centeredness, Community orientation, Evidence-based planning, Enhancing managerial thinking, Financial management and payment, and Effective surveillance. The table provides a comprehensive overview of the redesigned model’s structure and process categories.

Navigate back to Table 2..
